# Amyloid precursor like protein 1 in Idiopathic Normal Pressure hydrocephalus; expanding the knowledge of an altered amyloid metabolism

**DOI:** 10.1186/2045-8118-12-S1-O54

**Published:** 2015-09-18

**Authors:** A Jeppsson, M Höltta, H Zetterberg, K Blennow, C Wikkelsø, M Tullberg

**Affiliations:** 1 Hydrocephalus Research Unit, Institute of Neuroscience and Physiology, The Sahlgrenska Academy, University of Gothenburg, Sweden; 2 Clinical Neurochemistry Laboratory, Department of Psychiatry and Neurochemistry, Institute of Neuroscience and Physiology, the Sahlgrenska Academy, University of Gothenburg, Sweden

## Introduction

It has been shown previously that iNPH patients do exhibit suppressed levels of amyloid-β (Aβ) and the precursors soluble amyloid precursor protein α-, and β- (sAPPα, sAPPβ) in combination with depressed levels of tau proteins, both phosphorylated and total (t-tau, p-tau) and elevated levels of Neurofilament light protein (NFL). Expanding on this knowledge, we wanted to study if the changes seen in the amyloid processing pathways could be expanded to other fragments in the amyloid metabolism pathway.

## Methods

This retrospective study consists of 20 patients diagnosed with iNPH at the hydrocephalus unit at Sahlgrenska University hospital. 20 neurologically healthy individuals, undergoing knee surgery serves as healthy controls. All patients were examined clinically prior to surgery and at 6 month follow-up by the iNPH scale. Lumbar puncture was performed prior to surgery. Chemical analyses performed at the Clinical Neurochemistry Laboratory at the Sahlgrenska University hospital determined levels of NFL, Amyloid β isoform 38, 40, 42, soluble amyloid precursor protein alfa and beta, Amyloid precursor like protein 1 fragment 25, 27, 28 and YKL40.

## Results

We found a lowering of of sAPPα (0.50), sAPPβ (0.43) Aβ38 (0.45), Aβ40 (0.48), Aβ42 (0.32) and of APLP1b28 (0.88) in iNPH patients in comparison with healthy controls (HC) in combination with an elevation of APL1b25 (1.20) and APL1b27 (1.24) (concentration in iNPH/concentration in HC). NFL was elevated at a trend level, and YKL40 was suppressed, also at a trend level.

## Conclusions

Data on Ab38, -40, -42 and sAPPα and -β are confirmative of earlier results and might be a reflection of reduced periventricular metabolism and even disturbance of synaptic function. The increased levels of APL1b 25 and 27 might indicate a broader disturbance of the amyloid metabolism than previously thought. This new study might provide a piece in understanding the alteration of brain metabolism in patients with iNPH by the use of CSF biomarkers.
